# Pulmonary Hypertension Associated With Lung Disease: An Expert Review From the Cologne Conference

**DOI:** 10.1159/res/aepag019

**Published:** 2026-10-17

**Authors:** Silvia Ulrich, Katrin Milger, Michael Halank, Hans Klose, Tobias J. Lange, Hanno H. Leuchte, Esther J. Nossent, Hans-Jürgen Seyfarth, Oksana A. Shlobin, Dirk Skowasch, Michael Dreher, Juergen Behr

**Affiliations:** a Department of Pulmonology, University and University Hospital Zurich, Zurich, Switzerland; b Department of Pulmonology, University of Graz, Graz, Austria; c Department of Pulmonology, University of Dresden, Dresden, Germany; d Department of Pulmonology, University of Hamburg, Hamburg, Germany; e Internal Medicine II, Respiratory Medicine and Respiratory Care, Bad Reichenhall, Germany; f Internal Medicine, Pulmonology and Cardiology, Neuwittelsbach Hospital, Muenchen, Germany; g Department of Pulmonary Medicine, Pulmonary Hypertension and Interstitial Lung Disease, Amsterdam, The Netherlands; h Department of Pulmonology, Internal Medicine, Neurology and Dermatology, University Hospital Leipzig, Leipzig, Germany; i Pulmonary Hypertension Clinic, University of Virginia School of Medicine, Charlottesville, VA, United States; j Medical Clinic and Outpatient Clinic II, Heart Centre, University Hospital Bonn, Bonn, Germany; k Department of Respiratory Medicine and Internal Medicine Intensive Care, University Clinic Aachen, Aachen, Germany; l Internal and Respiratory Medicine, Ludwig Maximilian University of Munich, Munich, Germany

**Keywords:** Pulmonary hypertension, Chronic obstructive pulmonary disease, Interstitial lung disease, Pulmonary arterial hypertension, Lung phenotype

## Abstract

**Background:**

Pulmonary hypertension (PH) associated with chronic lung disease (CLD) (PH_lung) is a frequent complication of chronic obstructive pulmonary disease (COPD), interstitial lung disease, and other parenchymal lung disorders, and is associated with impaired exercise capacity, reduced quality of life and poor survival. Despite increasing recognition of its clinical importance, major challenges remain regarding early diagnosis, hemodynamic classification, and optimal treatment.

**Summary:**

This review summarizes current concepts of the diagnosis and management of PH_lung, with particular emphasis on multimodal screening strategies integrating pulmonary function testing, biomarkers, echocardiography, cardiopulmonary exercise testing, and thoracic imaging to improve patient selection for right heart catheterization. We discuss practical aspects of hemodynamic assessment, overlapping phenotypes, including connective tissue disease, sarcoidosis, and the emerging pulmonary vascular “lung phenotype,” and current evidence supporting targeted therapies with emphasis on patients’ pathways in real-world settings. While inhaled treprostinil has become the first approved treatment for PH associated with interstitial lung disease in some countries, no PH-specific therapy is currently recommended routinely for COPD-associated PH, highlighting the need for careful patient phenotyping and individualized management in expert centers. Earlier recognition, multidisciplinary collaboration, and continued translational and clinical research are essential to improve outcomes in this highly vulnerable patient population.

**Key Messages:**

Patients with PH_lung may present in pulmonary clinics with worsening CLD or are diagnosed in the work-up of PH. PH_lung is a severe condition that should be managed in expert centers with the possibility of advanced therapies, including transplantation and inclusion in clinical trials.

## Introduction

Chronic lung diseases (CLDs), including chronic obstructive pulmonary disease (COPD), the most common CLD worldwide, and interstitial lung disease (ILD), are frequently complicated by pulmonary hypertension (PH), which may ultimately lead to right heart failure in both advanced disease states and during acute exacerbations. A specific task force of the expert World Symposium on PH 2024 (6th WSPH) summarized, in a comprehensive review, current pathogenetic, diagnostic, clinical, and therapeutic knowledge and challenges of PH associated with CLD (PH_lung) [1]. Since then, several other documents have expanded on the topic of PH_lung [1–4]. This paper was written by a panel of experts from German-speaking countries who met in Cologne in June 2025 to discuss current topics and questions arising after the WSPH 2024, along with international experts with the goal of discussing challenging diagnostic and therapeutic decisions faced by clinicians in everyday practice and to identify the many areas of uncertainty in the field of PH_lung in order to direct future research.

The latest guidelines define PH by a mean pulmonary artery pressure (mPAP) of >20 mm Hg [5]. A pulmonary artery wedge pressure (PAWP) of ≤15 mm Hg in combination with a pulmonary vascular resistance (PVR) of >2 WU confers a hemodynamic diagnosis of precapillary PH [5, 6]. To define and potentially treat the underlying pathophysiology of PH_lung is a major challenge in everyday practice. PH_lung may not be solely precapillary due to the extensive overlap with left heart failure [7]. Hemodynamic assessment of PH may be challenging in patients with CLD due to marked intrathoracic pressure swings, particularly in emphysema with dynamic hyperinflation and in restrictive lung diseases with exaggerated negative inspiratory pressures [8, 9]. Other diagnostic challenges of PH_lung arise from the higher prevalence of pulmonary embolism in CLD [10], and the frequent overlap with comorbid conditions that may aggravate PH, such as an elevated hypoxic burden in PH-sleep apnea overlap [11–13], obesity or other hypoventilation syndromes, which may increase pulmonary artery pressure (PAP) due to arterial hypercapnia, pulmonary embolism, or other factors [14, 15]. PH_lung confers the worst prognosis compared to other PH diagnostic groups [16, 17]. However, it remains unknown whether treatment of PH in parenchymal lung diseases is associated with improvement in prognosis and long-term outcomes [18–21]. Although registry data, case reports and retrospective studies of PAH-approved targeted therapies in PH_lung suggest benefit, most prospective randomized-controlled trials (RCTs) largely failed to show beneficial effects or even showed harm in two RCTs [1, 22–26]. Thus, current guidelines suggest not treating PH_lung with PAH-approved drugs with the exception of individualized treatment in experienced PH centers in the presence of severe precapillary PH (PVR ≥5 WU) [5]. At presentation, PH may be suspected in a setting of established CLD due to clinical findings (Fig. 1, entrance gate lung disease), or CLD may be detected during work-up of patients with established PH (entrance gate PH in Fig. 1). In the following, we discuss current challenges in diagnosis, classification and management of these patients.

**Fig. 1.**
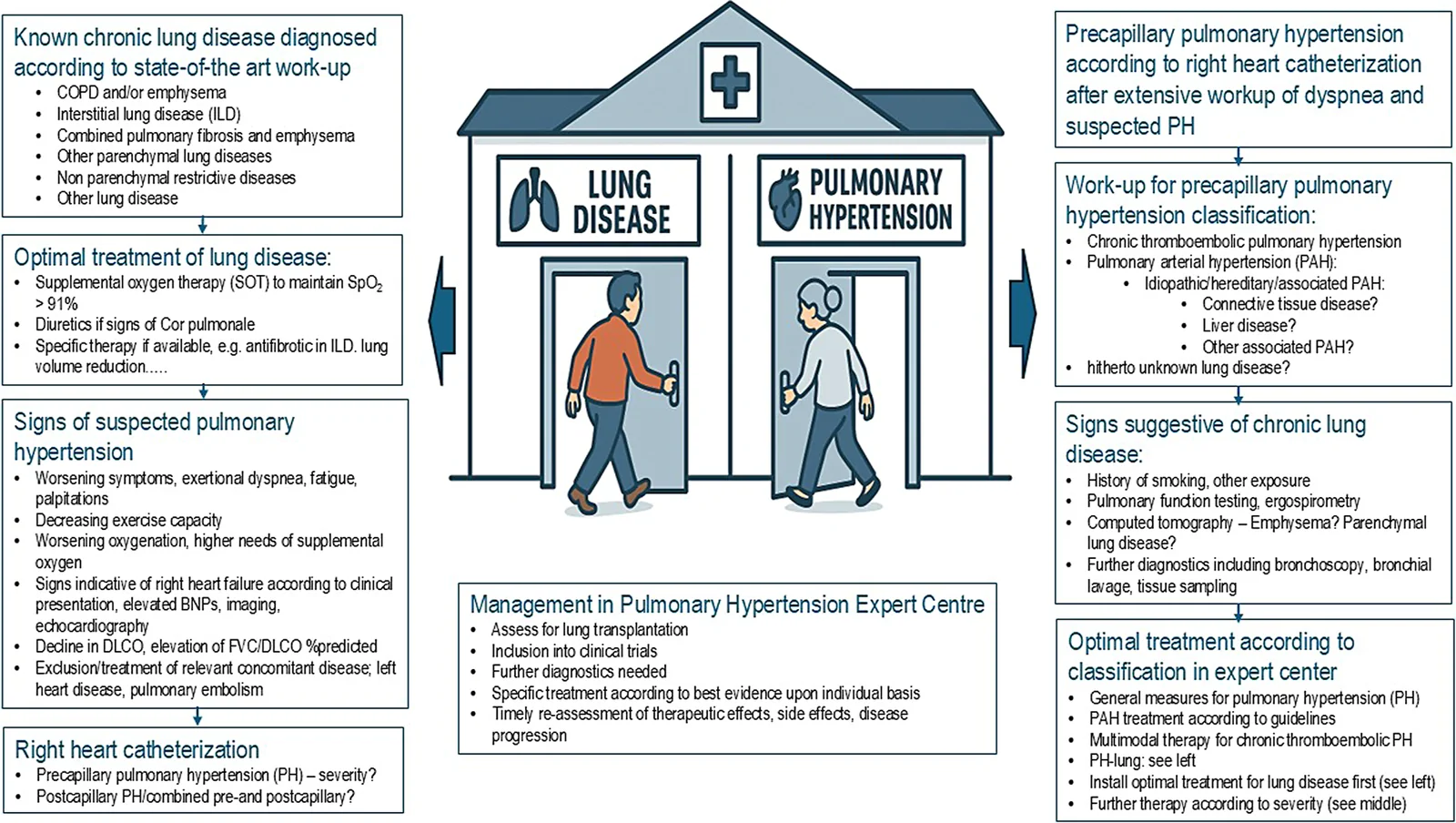
Patients’ clinical pathways for diagnosing and assessing pulmonary hypertension in patients with lung disease (right) and lung phenotypes in patients assessed to classify precapillary pulmonary hypertension (left).

### Patient Pathway Entrance Gate CLDs

#### Epidemiology of PH_lung in CLD and Pretest-Probability of PH

After the introduction of the new definition of PH, the prevalence of PH has increased significantly [27]. Lowering of the mPAP and PVR thresholds resulted in an increase in the prevalence of precapillary PH_lung up to 37 and 28% in PH_ILD and PH_COPD, respectively, although these prevalences will still largely depend on the setting of the population investigated (e.g., pretransplant vs. general hospital vs. outpatients vs. primary care cohorts) [1, 27–29]. The severity and type (pre- or post-capillary) of PH in CLD weakly correlates with the severity of the underlying CLD and is influenced by comorbidities and setting; for example, exacerbations of CLD aggravate PH. The high prevalence of CLD and herein lack of therapeutic consequences for many subtypes of PH_lung explains why not all patients with parenchymal lung disease and possible PH undergo a full diagnostic assessment. However, given recent advances in treatment of PH-ILD, easily obtainable screening tools are required to increase the pretest probability for hemodynamically diagnosed PH. The following section summarizes the most recent data on multimodal characteristics which may help to risk stratify CLD patients for the presence of PH.

*Disease Duration.* Patients with long-lasting CLD usually have more advanced disease, and thus, disease duration may be a factor to be considered [30]. However, the time point of diagnosis may significantly vary with patient-related factors, such as disease perception, socioeconomic status, and the health care system, thus all influencing perceived disease duration.

*Pulmonary Function Tests.* In general, advanced stages of CLDs and worse oxygenation carry an increased risk of PH. In COPD, dynamic hyperinflation only very weakly correlates with PAP and PVR [19, 20, 31]. Similarly, the correlation between lung volume measures and severity of PH in idiopathic pulmonary fibrosis (IPF) is weak [32]. A subset of COPD and ILD patients develops severe precapillary PH despite only mild-to-moderate abnormalities on pulmonary function testing and imaging. These patients are considered to represent a pulmonary vascular phenotype [33–38]. In patients with PH_COPD, the mortality risk increased by 4% with every decline in DLCO by 1% [39, 40]. A relatively small study in COPD revealed that the FVC/DLCO and FVC/DLCO/VA-ratios correlated with the presence of PH [41]. A recent analysis of patients with severe COPD undergoing evaluation for lung transplantation found that assessment of lung volumes by plethysmography may provide additional insights. Patients with severe PH_COPD had similar degrees of airflow limitation, but lower lung volumes than those with non-severe PH_COPD or no PH. Herein, PVR correlated negatively with residual volume [42]. Low DLCO also correlates with PH in ILD [43], especially when lower in relation to (more preserved) lung volumes; for example, an FVC/DLCO of >1.5 predicted PH in scleroderma irrespective of ILD [44], and an FVC/DLCO of >1.7 or TLC/DLCO of >1.67 were predictive of PH in ILD [43, 45]. Another study showed that patients with fibrosing ILD with exercise-induced PH had significant differences in the FVC/DLCO-ratio and diffusion (KCO and DLCO in % pred.) at rest [46]. In addition, patients with combined pulmonary fibrosis and emphysema (CPFE) seem to have even lower DLCO values, with the incidence and severity of PH higher as compared with ILD or COPD alone [47].

*Blood Gases.* In COPD and ILD, several studies reveal a strong correlation between resting and end-exercise partial pressure of oxygen (PaO_2_) and PH [48–50]. However, there are no universal PaO_2_ cutoff levels below which PH_lung can be diagnosed; therefore, this marker is mainly used in conjunction with other measures [51, 52]. Given the physiological effect of alveolar hypoxia on pulmonary pressures, right heart catheterization should not be performed during acute exacerbation of lung disease. In contrast to the widely known hypoxic pulmonary vasoconstriction, the medical community is less familiar with hypercapnic pulmonary vasoconstriction, which can play a major role in CLDs [53]. In obesity-hypoventilation syndrome and also several other CLDs, treatment of hypercapnia with noninvasive ventilation substantially improved PH [54]. In COPD but not ILD, higher partial pressure of carbon dioxide (PaCO_2_) correlated with increased PAP, and clear cutoffs to diagnose PH are still lacking [55]. In patients with CPFE PaO_2_ values may be even lower as compared to patients with fibrotic ILD or COPD patients which may at least partially explain higher prevalence of PH [47].

*ECG.* In COPD and ILD, like in other CLDs, ECG signs of right heart deviation and hypertrophy, right bundle branch block, S1Q3T3 pattern, T-wave inversion in precordial leads and prolonged QTc are all predictors of PH [56, 57]. On the other hand, the absence of those parameters does not exclude PH, and specific cut-offs of ECG signs of PH are not described for CLD. In a study separating PAH or chronic thromboembolic pulmonary hypertension from healthy individuals, ECG parameters alone could not reliably diagnose or exclude PH due to low sensitivity [58].

*BNP/NT-proBNP (referred to as BNPs).* Studies have shown a correlation of BNPs with disease severity, PAP and exacerbations in COPD and ILD [18, 59–61]. Albeit, BNPs alone lack sensitivity and specificity to diagnose PH in CLD, they are important components of combined diagnostic tools and important risk and follow-up markers. It should be recognized that BNPs are generally higher in left heart disease compared to PH and increase with age and impaired renal function (at least NT-proBNP). An elevation of BNP above the age-specific cut-off value was predictive of a mPAP of >35 mm Hg in patients with PH_lung [62].

*Echocardiography.* Transthoracic echocardiography (TTE) is the most important screening tool for PH in patients with dyspnoea with a high clinical probability of PH, including those with CLD. A cut-off for the tricuspid regurgitation velocity pressure gradient of 48.5 mm Hg, corresponding to a tricuspid regurgitation velocity of 3.5 m/s, correlated with high probability of PH in COPD patients [63], although appropriate windows to measure tricuspid regurgitation velocity may be more challenging in patients with CLDs [64]. A stepwise composite approach of various TTE parameters, including RV function, improved the diagnostic accuracy for PH in ILD [65].

*Cardiopulmonary Exercise Testing.* Cardiopulmonary exercise testing (CPET) parameters such as peakVO_2_ of ≤16.7 mL/min/kg (Youden‐Index [YI] = 0.79), petCO@AT of ≤34 mm Hg (YI = 0.67), and VE/VCO_2_@AT ≥30 (YI = 0.76) have a high potential to predict a diagnosis of PH [66, 67]. Similarly, in patients with CPFE, CPET parameters of AaDO_2_ at peak exercise, VE/VCO_2_ slope, peak P(a-ET)CO_2_, and peak VO_2_ showed high sensitivity and, when combined, high specificity to differentiate between patients with/without PH [68].CPET parameters may also help select patients with PH in a cohort of 41 patients with CPFE [68]. A cutoff value of 44 for VE/VCO_2_ slope had a sensitivity and specificity of 94.7 and 72.7%, while a cutoff value of 11 mm Hg for P(a-ET)CO_2_ in combination with peak AaDO2 of >60 mm Hg had a specificity and sensitivity of 95.5 and 84.2%, respectively. Combining peak AaDO_2_ of >60 mm Hg with peak VO_2_/body weight/min of <16.5 mL/kg/min provided a sensitivity and specificity of 100 and 95.5%, respectively.

*Imaging.* High-quality CT is of critical importance to assess severity of CLD. The most important CT-derived parameters associated with the presence of PH in CLD are pulmonary artery (PA) diameter of >29 mm, PA/aorta-ratio of >1, right-to-left ventricular(RV/LV) ratio of >1 and the presence of a contrast medium reflux into the inferior vena cava [69–71]. Intravascular ultrasound-assessed stiffness and area of wall thickness predicted PH in patients with COPD and IPF [55]. In addition, CT angiography can assess the presence of proximal pulmonary embolism [72]. Dual-energy CTA and SPECT-CT scans have high sensitivity for the presence of chronic thromboembolic disease [73]. Artificial intelligence-based quantitative CT assessment of pulmonary fibrosis independently predicts survival in patients with pulmonary hypertension, independent of age, PVR, and DLCO [74].

*Combined Parameters/Diagnostic Scores.* Given the limitations of any single diagnostic test to predict the presence of PH, a set of multimodal parametric scores showed better sensitivities and specificities to improve correlation with hemodynamically diagnosed PH_lung. The 6th WSPH document provides an informative table of different scoring systems, which mostly use a combination of clinical parameters, pulmonary function and exercise tests as well as BNPs or TTE measures (Fig. 1, 2) [1, 75].

**Fig. 2.**
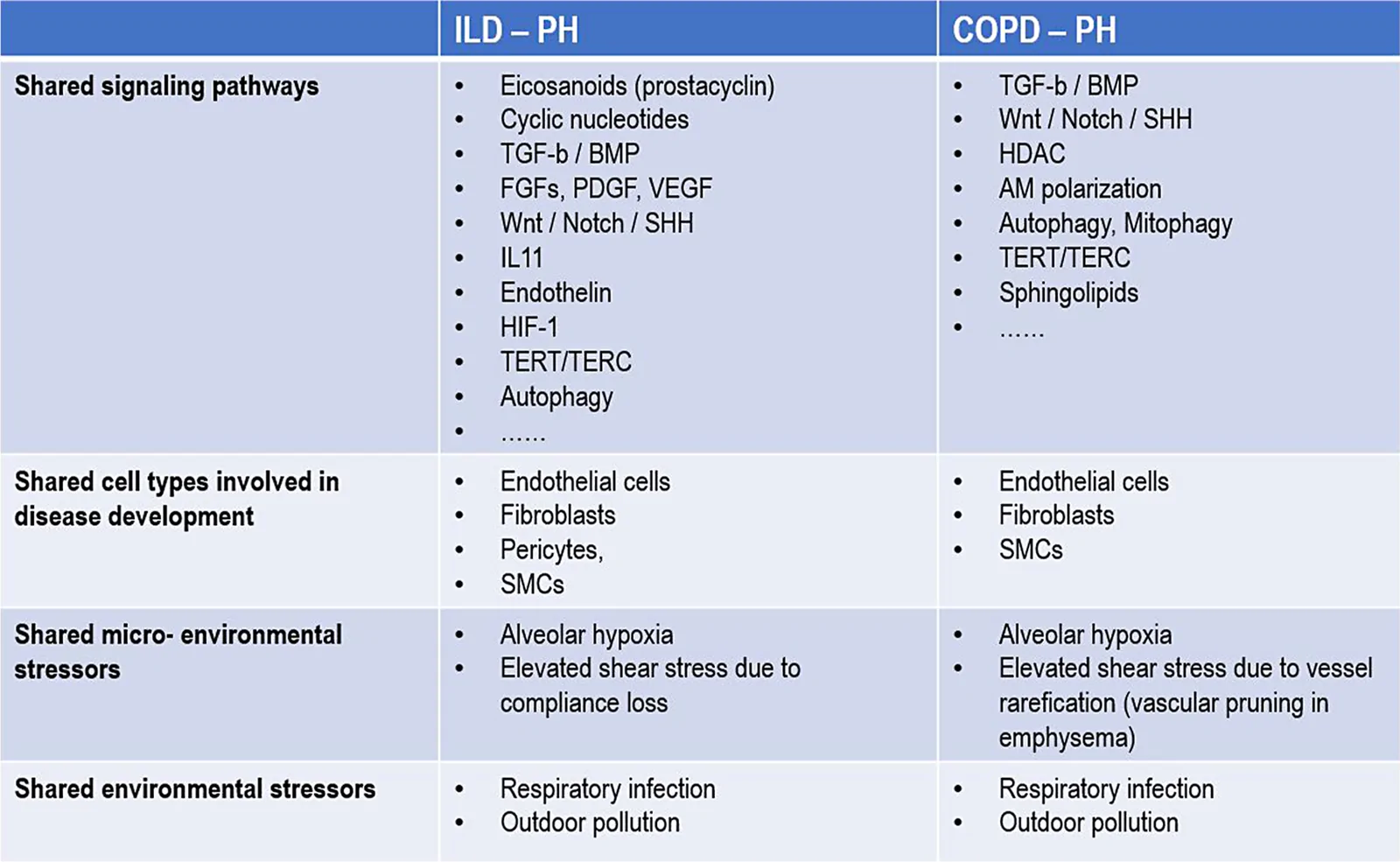
Potential pathomechanisms leading to pulmonary hypertension (PH) associated with lung disease (PH_lung) are shown separately for interstitial lung disease (ILD) and chronic obstructive pulmonary disease (COPD). TGF-β, transforming growth factor β; BMP, bone morphogenetic protein; FGF, fibroblast growth factors; PDGF, platelet-derived growth factor; VEGF, vascular endothelial growth factor; SHH, sonic hedgehog; IL11, interleukin 11; HIF-1, hypoxia-inducible factor1; TERT/TERC, telomerase reverse transcriptase/telomerase component RNA; SMCs, smooth muscle cells; HDAC, histone deacetylases.

#### Which Patient With CLD Reveals Signs of Severe PH and Should Undergo Further Investigation Using Right Heart Catheterization?

As described above, there is no single stand-alone parameter to indicate which patient should undergo a right heart catheterization (RHC), but rather a combination of symptomatic worsening, exercise testing results, PFTs, biomarkers, TTE findings and imaging [1, 76]. Studies like PHINDER are on the way to investigate this topic [77]. Moreover, the patient's condition and expectations as a whole and availability of treatment options need to be taken into consideration, and close collaboration with expert centers in PH and advanced lung diseases is recommended.

Indications for RHC:

1. Classification to direct treatment (precapillary PH, heart failure, etc.).
2. Evaluation for clinical trials.
3. Prognostication.
4. Hemodynamic assessment before interventions such as endoscopic lung reduction, LVRS, and lung transplantation.

#### Which Patient With CLD Should Not Undergo RHC?

Given the invasive nature of RHC, individual decision-making needs to be used to determine whether the results of an RHC would change the therapeutic management.

In general, patients with CLD and acute hypoxemia and/or hypercapnia should not undergo RHC until acute illness has resolved due to the above-discussed effects of hypoxemia and hypercapnia on pulmonary pressure and transient cor pulmonale physiology. In rare situations, acutely ill patients need to undergo RHC for either lung transplant work-up.

These patients with exacerbated CLD revealing severe hypoxemia and/or hypercapnia should first be treated with supplemental oxygen therapy, including high-flow nasal oxygen (HFN, supplemental oxygen therapy [SOT]) or noninvasive ventilation, in order to achieve sufficient blood oxygenation and/or effective PaCO_2_ reduction, and have concomitant aggravating diseases ruled out, for example, acute pulmonary embolism. Indication of an RHC should then be made in an expert center in a stable condition.

#### How Should the RHC Be Performed in PH_lung?

Hemodynamic measures during RHC are generally performed in the supine resting position, as recommended for all forms of PH [9]. Due to potentially considerable intrathoracic pressure swings in COPD with emphysema in particular, but also other CLDs, it is recommended to average pressure tracing over several respiratory cycles [78]. Patients with both COPD and ILD frequently have cardiovascular comorbidities, and careful assessment of the risk of heart failure needs to be performed clinically and assessed by echocardiography. In both diseases, up to 30% of patients have concomitant heart failure with preserved ejection fraction [7], thus making accurate measurement of PAWP particularly important. In uncertain cases, direct pressure tracing in the left ventricle by insertion of a pigtail catheter should be considered. Most experts recommend that hemodynamic measurements should be performed during euvolemic and normoxemic conditions, and SOT should be used to achieve SpO_2_ if possible around or above 90%, but data on this approach are scarce. A test with high-dose oxygen is used by some experts to investigate the degree of remaining pulmonary vasoreactivity, but the clinical value of this procedure is not clear, and further studies are warranted before this approach could be recommended. One study revealed that a reduction of the heart rate below 72 bpm after application of oxygen was associated with better long-term outcome, potentially reflecting a reduction in sympathetic overexcitation by supplemental oxygen [79]. Acute vasoreactivity testing (AVT) is not recommended in PH_lung due to the paucity of studies showing a predictive value on outcome or treatment response [80]. One study investigating 48 CLD patients with inhaled NO did, however, reveal that acute responders (>15%PVR reduction) were showing fewer symptoms, lower BNP and improved hemodynamics after a median follow-up time of 4 months [81, 82]. Other experts consider an AVT with a single dose of the rapid-onset PDE-5 inhibitor sildenafil as helpful for safety estimates to unravel potential side effects of worsening hypoxemia. However, to the best of our knowledge, this strategy has never been investigated prospectively in terms of safety or efficacy. Exercise RHC is currently used in patients with CLD in research settings. One study showed that exercise pulmonary hemodynamics also revealed a prognostic value in patients with COPD [83].

#### Which PAH-Targeted Treatments Are Currently Available for PH_lung?

General measures recommended for PAH also apply to PH_lung [5, 84]. These include diuretics, treatment of comorbidities, SOT in the presence of hypoxemia, noninvasive ventilation in the presence of hypercapnia, guided exercise/rehabilitation programs, psychological/social support and palliative care to manage dyspnea [85]. In COPD specifically, and according to clinical practice also in ILD, domiciliary SOT is recommended for at least 15 h in CLD with severe hypoxemia below a threshold of 8 kPA (60 mm Hg) in the presence of PH, although prospective studies are lacking and the benefit in nonsevere hypoxemia is questionable [86, 87]. High-dose oxygen therapy in COPD increases exercise capacity in terms of power output in incremental and time in constant work-rate exercise [88], but whether low-dose oxygen therapy via nasal cannula provided by portable devices is beneficial to prolong exercise capacity or daily activity is unknown.

*PAH-Targeted Therapies in COPD.* Currently, there is no PAH-targeted medication approved worldwide for the treatment of precapillary PH in association with COPD.

According to the ESC/ERS guidelines and the 7th WSPH, no PAH-targeted therapy is recommended for COPD patients with non-severe PH (defined as PVR ≤ 5 WU) [1, 5]. Chronic obstructive pulmonary disease patients with severe PH, currently defined according to guidelines as PVR > 5 WU, should be referred to a PH center for individualized treatment strategy [1, 5]. Before starting any treatment with PH-specific drugs, PH_COPD patients should individualized treatment strategy undergo phenotyping with the goal to preselect patients who are most likely to benefit from therapy. Even if there is no evidence-based approach, most experts consider patients with higher PVR and pathophysiological features of the “COPD-pulmonary vascular phenotype” (severe precapillary PH despite only moderate airflow limitation, markedly reduced DLCO, and poor clinical outcomes) [1, 18].

*RCTs in PH_COPD.* PERFECT is the largest prospective, placebo-controlled, randomized study to date in patients with hemodynamically confirmed PH_COPD patients treated with inhaled treprostinil (*n* = 66). The primary endpoint of the study was the placebo-adjusted change in the 6MWT distance after 12 weeks of therapy [22]. Inclusion criteria were mPAP of ≥30 mm Hg, PVR of ≥4 WU, and PAWP of ≤15 mm Hg. Since the mean FEV1% predicted was greater than 40%, it can be postulated that a proportion of patients with a pulmonary vascular phenotype were included. This study was terminated prematurely following an interim analysis due to an increased incidence of serious adverse events, an increased rate of drug discontinuations, and a potentially increased risk of mortality with the study drug compared to placebo. In addition, there was no evidence of a positive effect of inhaled treprostinil on exercise capacity. The change in the 6-minute walking distance (6MWD) was −4.5 m in the treprostinil group and −5.1 m in the placebo group. One of the shortcomings of the study is the absence of centrally analyzed chest CT imaging. A post hoc analysis showed that patients with a DLCO of <25% were less likely to benefit and more likely to be harmed by treatment. The study was also analyzed for patients who may potentially respond positively to treatment with inhaled treprostinil. Treatment response was defined as an increase in 6MWD of ≥15% with a decrease in NT-pro-BNP of ≥20%. Only patients with mPAP of ≥40 mm Hg, FEV1 of >40% predicted, and DLCO of >25% predicted were thought to be potential therapy responders. Patients with an RV/TLC ratio of >60% did not respond to therapy [89].

The second largest prospective, placebo-controlled, randomized multicenter study in COPD-PH patients confirmed by RHC was conducted with tadalafil (*n* = 28). Patients with mPAP of >25 mm Hg, PVR of >2.5 WU, and PAWP of ≤18 mm Hg were included [90]. The primary endpoint of the study was a change in the 6MWD after 1 year of therapy, and secondary endpoints included changes in mPAP and PVR after 6 months of therapy. Due to a very high dropout rate, analysis of the primary endpoint after 1 year was only possible in 9 patients in the placebo arm and in only 15 patients in the tadalafil arm. The PVR in the tadalafil group was 4.2 (2.8–5.9) WU at baseline, and in the placebo group 3.9 (3.1–5.5) WU (NS). FEV1 was 52 ± 21% predicted and 50 ± 32% predicted in the treatment and placebo groups, respectively. No significant difference between the treatment and placebo arms was found in this study for either the primary endpoint or the secondary endpoints mentioned above. This could be due to the more liberal hemodynamic inclusion criteria, asymmetrical patient recruitment at the study centers, randomization imbalance, and insufficient power to detect a tadalafil effect on 6MWD and/or hemodynamic improvement. Interestingly, a significant difference (*p* = 0.02) in the improvement of the University of California San Diego shortness of breath questionnaire was reported between the two treatment groups in favor of tadalafil.

The third largest prospective, placebo-controlled, randomized multicenter study (SPERIC-1) in hemodynamically confirmed PH_COPD patients was to assess the effect of sildenafil on PVR (*n* = 28) [91]. The PVR of the sildenafil treated group was 7.0 ± 2.6 WU and 6.3 ± 3.1 WU in the placebo group (NS). There was no difference in the FEV1 between the sildenafil and placebo groups (54 ± 22% and 48 ± 25% predicted, respectively [NS]), comparable to the tadalafil study. The Tiffenau index in the sildenafil (52 ± 13%) and the tadalafil (53 ± 8%) groups were also comparable. Sildenafil (*n* = 18) led to a significant reduction in PVR (Δmean difference from placebo −1.4 WU; 95% CI ≤ −0.05; *p* = 0.04) after 16 weeks of therapy compared to placebo (*n* = 10). Furthermore, sildenafil led to an improvement in placebo-corrected secondary endpoints such as the BODE index (*p* = 0.02), quality of life (SF-36 questionnaire; *p* = 0.02) and an improvement in the DLCO % predicted. The 6MWD in the sildenafil arm was numerically but not statistically significant (Δmean +8.1 m vs. Δmean −11.2 m, respectively).

In other previously published randomized, double-blind, placebo-controlled studies with PH-specific drugs, right heart catheterization was not mandatory for the diagnosis of PH, so these studies will not be discussed further.

*Non-Randomized and Retrospective Trials in PH_COPD.* A retrospective analysis of a large global meta-registry (Pulmonary Vascular Research Institute GoDeep Meta-Registry) investigated the potential effect of sildenafil therapy in patients with hemodynamically confirmed PH_COPD [24]. Treated patients (*n* = 418) had significantly better transplant-free survival (*p* < 0.001) compared to untreated patients (*n* = 357). This was true for both patients with severe PH (PVR > 5 WU) and non-severe PH. However, in patients with non-severe PH, the survival benefit was only evident after 2 years of therapy. The median FEV1 in treated patients with severe PH was 58 (IQR, 40–76) % predicted and 54 (IQR, 39–66) in those with non-severe PH. The authors recommended prospective randomized studies to validate the therapeutic effect of PDE-5 inhibitors and suggested that therapy with a PDE-5 inhibitor may be considered in patients with PH_COPD on an individual basis.

In summary, PH_COPD patients should be referred to PH centers so that they can be included in ongoing drug trials. If there are no trials available, the use of a PDE 5 inhibitor may be considered on an individual basis in cases of severe precapillary PH. In addition, inclusion in a registry should be strongly encouraged. Ongoing trials include the following.

A phase 3 study is currently investigating the efficacy of tadalafil (TADA-PHILD, NCT01862536), and a phase 2 study is investigating the efficacy of an inhalable soluble guanylate cyclase stimulator MK-5475 (INSIGNA-PH-COPD, NCT05612035).

In both studies, the 6MWD difference is the primary endpoint. Although it is doubtful whether this endpoint is the best for PH_COPD studies, the6MWD outcome is accepted by regulatory agencies, and many other endpoints such as composite or patient-reported outcomes still need validation.

*PH-targeted Drug Therapy in ILD.* For the treatment of PH_ILD, inhaled treprostinil is the only pharmacological therapy currently approved in the USA, Israel, Chile, the Dominican Republic, and Argentina. An EMA approval is lacking to date, and it is under consideration in Japan. A double-blind, placebo-controlled prospective clinical trial (INCREASE) investigated the efficacy and safety of inhaled treprostinil in patients with various forms of fibrotic ILD, CPFE and pulmonary hypertension hemodynamically defined as mPAP of ≥25 mm Hg, PAPW of ≤15 mm Hg and PVR >3 WU [92]. The primary endpoint, 6MWD, showed a significant placebo-corrected improvement of 31.1 m at 16 weeks. In addition, patients treated with treprostinil experienced fewer clinical deteriorations and showed a significant decline in serum NT-pro-BNP. Consequently, the 7th recent PH guidelines and WSPH recommend considering treatment with inhaled treprostinil for PH_ILD [1, 5]. Of interest, patients with CPFE were also included in the increase trial, but available evidence does not support a statistically significant or clinically robust benefit from inhaled treprostinil in this subgroup [93, 94]. Recently, a phase II trial of treprostinil palmitate showed that this easily administrable once-daily inhalation formula revealed no new safety concerns and showed trends toward improvement in 6MWD and NT-proBNP, so this drug is currently being investigated in a phase III trial [95].

Utility of PDE5 inhibitors in PH_ILD is controversial. Placebo-controlled clinical trials investigating PDE-5 inhibitors in IPF populations enriched for PH but without hemodynamic confirmation were negative for 6MWD as the primary endpoint but showed some positive signals for secondary endpoints like DLCO, dyspnea, and SpO_2_ [96]. On the other hand, there is registry data that suggests similar hemodynamic benefits of PH medications, mostly PDE-5 inhibitors, as observed in PAH [97–99]. Another randomized controlled study testing inhaled nitric oxide on top of LTOT was also negative, but this was not in a hemodynamically defined PH_lung population [100]. Based on these heterogeneous data, the current ERS/ESC guideline recommends consideration of treatment with PDE-5 inhibitors only in severe PH_ILD (i.e., PVR >5 WU) (recommendation class IIb, level C). A large meta-registry analysis across 940 hemodynamically characterized PH_ILD patients published after the ESC/ERS and 7th WSPH documents suggested a positive effect of PDE-5 inhibitors on overall survival in patients with PH [101].

A clinical phase 2b trial of PH_ILD evaluating riociguat versus placebo in idiopathic interstitial pneumonia patients was terminated early for safety reasons and potential harm; another clinical trial evaluating ambrisentan versus placebo in IPF was also terminated early due to futility and potential harm, but the presence or absence of PH was not an inclusion criterion [25, 26]. The ERS/ESC guideline recommends against use of these drugs in particular (class III recommendation, level B) [5].

Ongoing clinical trials in PH_ILD include inhaled treprostinil-palmitil (once-daily powder inhalation) [102], other inhaled treprostinil formulas, inhaled mosliciguat and others.

### Patient Pathway Entrance Gate Evaluation of PH

Every patient diagnosed with precapillary PH should be evaluated for underlying lung disease. Importantly, PAH may coexist with mild COPD or other mild CLD, and the presence of limited parenchymal abnormalities should not automatically preclude a diagnosis of PAH. However, even in the absence of overt underlying lung disease, it has become clear over the last decades that a subtype of patients which are currently classified as PAH according to precapillary hemodynamics and lack of apparent and diagnosed CLD according to pulmonary function testing and imaging reveal features pointing towards a hitherto not diagnosed CLD, especially a history of smoking and a markedly reduced DLCO [103–105]. This so-called “lung phenotype” characterized by older age, predominant male sex, smoking history, cardiovascular comorbidities, and severely reduced DLCO (mostly ≤45% pred.) but with preserved spirometry and minimal or no overt parenchymal lung disease on imaging, is increasingly identified in several PAH registries [105–109]. Patients with this “lung phenotype” exhibit a non-plexiform vasculopathy characterized by severe microvascular remodeling and pulmonary capillary rarefaction, more closely resembling PH associated with CLD, particularly PH_COPD, than classical idiopathic PAH. This concept is further supported by advanced imaging studies demonstrating early diffuse emphysema and pulmonary capillary loss on hyperpolarized xenon (^129Xe) MRI in patients with idiopathic PAH and lowered DLCO, as well as by evidence that pulmonary microvascular dysfunction is already present in mild COPD before extensive emphysematous destruction becomes apparent [110, 111]. However, these patients were included in RCTs investigating the effectiveness of various PAH-targeted drugs and according to available registry data, they respond to PAH-targeted therapies, although to a lesser extent than patients with classical PAH, and reveal a worse prognosis [112–114]. It would be of interest to have an individual patient data meta-analysis specifically evaluating the effect of PAH-targeted drug therapy in patients with this lung phenotype from existing trials and to predefine these subgroup analyses in current and future RCTs in the field of PAH.

### Specific Aspects and Diseases Which May Lead to Overlapping PH-Phenotypes

*Connective Tissue Disease-Associated CLD.* Connective tissue diseases (CTDs) are multisystemic diseases, which may predominantly involve lung parenchyma but also may affect cardiac and pulmonary vasculature and extra-thoracic organs, such as the liver. Thus, PH in CTD may be very hard to classify: if PH is clearly predominant and fibrosis mild, we classify as PAH; if fibrosis is predominant, we classify as PH_lung, but mixed situations clearly exist [5, 6]. Right heart catheterization is recommended in all cases of suspected PAH associated with CTD. In addition, annual screening for PAH is recommended in patients with systemic sclerosis and should also be considered in those with systemic sclerosis overlap syndromes.

*Sarcoidosis-Associated CLD.* Pulmonary hypertension is found at an above-average rate in patients with sarcoidosis (sarcoidosis-associated PH, SAPH) [115], with a reported prevalence of 6–20% [116]. According to the current classification of PH (WHSP ref.), SAPH falls into group 5 PH, as it can be caused by factors such as left ventricular dysfunction, direct vascular compression by enlarged lymph nodes, and primary granulomatous vasculopathy (although rare), in addition to fibrotic lung disease, which is the most common etiology [116]. Pulmonary hypertension confer high morbidity and worse survival in patients with sarcoidosis compared to those without PH [117–119]. One report suggested that in patients with severe SAPH mostly due to fibrotic lung disease, PH treatment was associated with improved short-term pulmonary hemodynamics, but without improving exercise capacity measured with 6MWD [120]. A double-blind, placebo-controlled randomized trial demonstrated that bosentan, an endothelin receptor antagonist, significantly improved pulmonary hemodynamics (mean pulmonary artery pressure and pulmonary vascular resistance) in SAPH, but did not improve exercise capacity or quality of life over 16 weeks [121]. A double-blind, placebo-controlled trial randomized patients with sarcoidosis-associated PH confirmed by RHC to riociguat or placebo for 1 year. Riociguat was associated with a significant delay in time to clinical worsening and improvement in 6MWD compared to placebo, with no significant adverse events reported [122]. More studies are needed to assess which phenotypes of SAPH patients may respond to therapy and the utility of various PH drugs [123].

### Lung Transplantation as Treatment Option in PH_lung

Lung transplantation is an established therapy for selected patients with advanced CLD and PH, when maximal medical and surgical therapies have failed, and prognosis is poor [124]. The presence of severe PH in CLD is associated with worse functional status, increased morbidity, and higher mortality [125], and it often serves as a marker for disease severity and transplant urgency [30].

For COPD, referral is considered when FEV_1_ falls below 25% predicted [126], with hypoxemia, hypercapnia, or frequent exacerbations despite optimal therapy. Patients with progressive fibrotic-ILD should be made familiar with the lung transplant option at the time of diagnosis and undergo timely evaluation in case of any worsening, due to their dismal prognosis, to be prepared in case of accelerated and potentially unpredictable disease progression and to prevent waitlist mortality.

The main contraindications for lung transplantation are divided into absolute and relative categories [126]. Absolute contraindications include: lack of patient willingness or acceptance, active malignancy with high risk of recurrence or death, recent acute coronary syndrome or stroke, liver cirrhosis with portal hypertension or synthetic dysfunction, acute liver or kidney failure with low likelihood of recovery, septic shock, active extrapulmonary or disseminated infection, HIV infection with detectable viral load, limited functional status with poor rehabilitation potential, progressive cognitive impairment, repeated nonadherence, active substance use or dependence (including tobacco, vaping, marijuana, or intravenous drugs), and other severe uncontrolled medical conditions expected to limit survival after transplantation. Relative contraindications include the following: age >65 years, severe coronary artery disease requiring bypass at transplantation, reduced left ventricular ejection fraction of <40%, substantial cerebrovascular disease, severe esophageal dysmotility, untreatable hematologic disorders, short telomeres (<1%) and/or telomerase mutations, BMI of >35 or <16 kg/m^2^, limited functional status with potential for rehabilitation, psychiatric or cognitive conditions interfering with adherence, unreliable support system, lack of understanding of disease or transplantation, certain resistant infections (e.g., *Mycobacterium abscessus*, *Burkholderia cenocepacia*), hepatitis B or C with detectable viral load and liver fibrosis, chest wall or spinal deformity causing restriction, and recent transplantation (<1 year). Multiple risk factors are considered multiplicative in increasing risk, and modifiable conditions should be addressed when possible.

*Double versus Single Lung Transplantation.* While double-lung transplantation (DLTx) is clearly indicated in patients with PAH, the choice between single-lung transplantation (SLTx) and DLTx in CLD with PH remains less defined and should also account for organ availability and the imperative to optimize donor lung utilization across the recipient population. For patients with COPD DLTx is associated with longer survival, particularly in patients under 60 years of age [127–129]. Also in ILD, a meta-analysis including 17,872 patients showed no difference in long-term survival between SLTx and DLTx [129], as discussed in detail elsewhere [130]. Double-lung transplantation is often preferred in patients with severe PH_lung, particularly when mean PAP exceeds 40 mm Hg, based on retrospective registry data suggesting inferior outcomes with SLTx [131]. However, these comparisons are limited by heterogeneous populations and lack adjustment for underlying disease [132].

In summary, early referral for lung transplantation is essential in eligible patients with PH_lung to allow for timely evaluation, optimization of modifiable risk factors, and to reduce wait-list mortality, as delays in referral are associated with worse outcomes and missed opportunities for intervention [133]. Recent data highlight that current referral rates remain unacceptably low and often occur too late even at PAH expert centers, underscoring the need for increased awareness and proactive referral practices [134].

### What Are Future Directions of Research in PH_lung?

Although PH is common in CLD, not all patients with a comparable severity of lung disease develop PH. Currently, we lack convincing data to explain why some CLD patients, even within the very same disease category, develop PH_lung, whereas others do not. The current pathomechanistic concepts are illustrated in Figure 2 and cover a wider spectrum of possible explanations. Future research activities will help to identify robust biomarkers indicating the susceptibility of the pulmonary vasculature to undergo vascular remodeling and development of PH_lung and thereby to identify CLD patients being at risk of developing PH_lung. Upcoming imaging techniques such as quantitative CT measures of pulmonary vascular volume distribution or MRI measuring of pulmonary arterial stiffness may help to differentiate PH_lung patients with a treatable pulmonary vascular component from the others [135, 136]. The development of PH_lung clearly results in a worse prognosis. Hence, having in mind the frequency of PH_lung in CLD, and the high prevalence of CLD as a whole, it appears mandatory to intensify research efforts to identify effective treatments for PH_lung. Moreover, future therapeutic trials should investigate whether treatment of PH_lung affects quality of life and disease progression, not only 6MWD. Finally, it will be important to determine whether treatment of PH affects the pathogenesis of the underlying disease, as suggested by the data of inhaled treprostinil in IPF [137]. Similarly, there is still a lot of uncertainty about the diagnosis, classification and best management options for patients with the “lung phenotype.” An ERS-task force is working on a statement about definitions, state-of-the art knowledge and future perspectives for idiopathic PAH and comorbidities.

Performing RCTs in this field of PH_lung, or also “lung phenotype,” is challenging for several reasons. First, the underlying diseases are heterogeneous, and the patient population is highly symptomatic and often comorbid, which makes enrolment into trials difficult due to logistical and inclusion criteria, and because of the potentially rapid disease progression. Potentially novel and adaptive trial designs may help to overcome some of these problems. Whether new antifibrotic and anti-inflammatory drugs like nerandomilast, which showed positive effects in IPF and PPF populations, will also have an impact on pulmonary circulation needs to be evaluated [138, 139]. Upcoming studies using a variety of drugs, many of which are inhaled, will hopefully provide promising new approaches to the treatment of PH_ILD and potentially PH_COPD [140].

## Conclusions

In conclusion, PH_lung is a highly prevalent and relevant condition, and a considerable part of patients fulfilling current diagnostic criteria for PAH have features of CLD and share the dismal prognosis of PH_lung. The diagnosis of PH_lung is often impeded or postponed due to the lack of awareness among medical professionals, which is leading to underdiagnosis. This is primarily due to the prevailing opinion among treating physicians that there are no effective therapeutic interventions in this context. However, data from registries and clinical trials demonstrate that a subset of patients may benefit from specific therapeutic interventions. It is imperative to establish a close collaborative relationship between international experts, industry and other stakeholders to enhance pathophysiological knowledge and develop effective therapies for this highly disabled PH_lung patient population.

## Conflict of Interest Statement

S.U. receives research grants from the Swiss National Science Foundation, Zurich and Swiss Lung League and EMDO Foundation and grants, travel support and consultancy fees from Orpha Swiss, Janssen SA, MSD SA, Gebro SA, Ideogenand, and AstraZeneca, all unrelated to the present work. Silvia Ulrich was a member of the journal’s Editorial Board at the time of submission. K.M. received speaker and/or advisory fees from AstraZeneca, Celltrion, Chiesi, Ferrer, Insmed, GSK, Janssen, Orion, Sanofi. M.H. received Honoraria for lectures or for participation of advisory boards: AOP Pharma Germany, Janssen-Cilag, and MSD. Support for attending meetings and/or travel: AOP Pharma Germany and Janssen-Cilag. H.K. has received speaker fees and honoraria for consultations from Actelion, Bayer, GlaxoSmithKline, Janssen, MSD, Novartis, Pfizer and United Therapeutics. T.J.L. reports personal fees for lectures from Johnson & Johnson, MSD, Boehringer-Ingelheim, AstraZeneca, Pfizer, BMS, Ferrer, and Daiichi-Sankyo; support for attending meetings and/or travel from AstraZeneca, Boehringer-Ingelheim and AOP Orphan Pharmaceuticals, personal fees for participation on a data safety monitoring board or advisory board from AstraZeneca, Johnson & Johnson, MSD, and Boehringer-Ingelheim, and personal fees as a member of the scientific committee for a registry study conducted by Actelion (Johnson & Johnson). H.H.L. received speaker and advisory fees from AOP, AstraZeneca, Bayer, Boehringer Ingelheim, Gossamer Bio, Insmed, Johnson & Johnson, Liquidia, MSD, Pfizer, Roivant. E.J.N. reports research grants or institutional research support from Boehringer Ingelheim B.V., AbbVie, and PEXAS (P4O2 ILD extension trial). She has received honoraria for lectures and educational activities from Ferrer, MSD, Boehringer Ingelheim, and BMS. She has served on advisory boards for MSD and Gossamer Bio/Chiesi. H.J.S. received speaker and advisory fees from Johnson & Johnson and MSD. O.S. is on steering committees for the following companies: AllRockBio, Gossamer, Insmed and consults for the following companies: United Therapeutics, Merck. O.S. is also a speaker for United Therapeutics. D.S. received speaker and advisory fees from AstraZeneca, Boehringer Ingelheim, Chiesi, GSK, Johnson & Johnson, MSD, Roche, Sanofi. M.D. receives fees for speaking from Actelion, Aerogen, Astra Zeneca, Bayer, Berlin Chemie, Boehringer, Chiesi, GSK, Hamilton, Heinen und Löwenstein, Insmed, Inogen, Intermune, Linde, Novartis, Pohl-Boskamp, Pfizer, Philips Respironics, ResMed, Roche, Sanofi, Weinmann and fees for consulting from Almirall, Berlin Chemie, Boehringer, GSK, Hamilton, Inogen, Linde, Novartis, Pohl-Boskamp, Pfizer, Philips Respironics, ResMed, Roche, Sanofi and research grants from BMBF, DFG, GB-A, Land NRW, Philips Respironics, ResMed, Löwenstein Medical. J.B. reports grants from Atemweg-Foundation, BMBF, DFG and Boehringer Ingelheim; and consultancy fees from AstraZeneca, Biogen, BMS, Boehringer Ingelheim, Ferrer, Galapagos, Gossamer Bio, Johnson & Johnson, Novartis, Pliant, Pulmovant, Roche and Sanofi-Genzyme.

## Funding Sources

We had no external funding for this review paper.

## Author Contributions

S.U. did the first outline of the paper and drafted the main section after extensive discussion of the content in Cologne. K.M., M.H., H.K., T.J.L., H.H.L., E.J.N., H.J.S., O.S., D.S., M.D., and J.B. contributed to writing of sub-sections and reviewed the entire content of the review. All authors approved the final manuscript.
